# Reducing the risk of criminal exploitation using multi-systemic therapy (the RESET Study): study protocol for a feasibility study and process evaluation

**DOI:** 10.1186/s40814-023-01409-9

**Published:** 2023-11-27

**Authors:** N. K. Hayden, S. Flynn, F. Blumenfeld, R. P. Hastings, K. M. Gray, S. Cullen, M. A. Cullen, P. E. Langdon

**Affiliations:** 1 Centre for Research in Intellectual and Developmental Disabilities (CIDD), University of Warwick, Coventry, UK; 2 School of Health and Social Care, University of Essex, Colchester, UK; 3 Department of Psychiatry, School of Clinical Sciences at Monash Health, Monash University, Melbourne, Australia; 4 Coventry and Warwickshire Partnership NHS Trust, Coventry, UK; 5 Herefordshire and Worcestershire Health and Care NHS Trust, Worcester, UK

**Keywords:** Youth, Children, Social and behavioral problems, Family, Criminal exploitation, Gangs, Multisystemic therapy

## Abstract

**Background:**

Child criminal exploitation is a form of child abuse that poses a serious risk to the welfare, safety, and wellbeing of young people. Multisystemic therapy (MST) is an intensive family and community-based intervention for young people with anti-social behavioral problems, many of whom will be at risk of criminal exploitation. This protocol describes a pilot feasibility study and process evaluation, designed to examine MST for children at risk of criminal exploitation.

**Methods:**

This pilot feasibility study and process evaluation involves two phases with associated subphases: phase 1.1 involved the collaborative refinement of the logic model adapting MST for children at risk of criminal exploitation; phase 1.2 involved pre-pilot interviews with MST therapists, families, and young people; phase 2.1 is a pilot modeling study of MST for children at risk of criminal exploitation, and; Phase 2.2 is a process evaluation that will involve interviewing stakeholders, MST therapists and employees, families, and young people. The dataset for the process evaluation will include questionnaires completed by parents and young people at baseline, mid-treatment, end of treatment, and 6 months after treatment. We will supplement these data with participant-level data linkage from MST sites and services.

**Results:**

Accrual to the pilot stage of this project opened on 6th August 2021 and is due to close on 31st May 2022. We aim to publish the results of this feasibility study and process evaluation in 2023.

**Conclusions:**

The results of this feasibility study and process evaluation will inform the decision as to whether it is advisable to progress to a pilot clinical trial of MST for children at risk of criminal exploitation.

**Trial registration:**

Trial registration: ISRCTN registry, ISRCTN16164816 on 25th January 2021—https://doi.org/10.1186/ISRCTN16164816.

## Background

Child criminal exploitation (CCE) is where ‘an individual or group takes advantage of an imbalance of power to coerce, control, manipulate, or deceive a child or young person under the age of 18 into any criminal activity in exchange for something the victim needs or wants, for the financial or other advantage of the perpetrator or facilitator, or through violence or the threat of violence’ [1]. CCE is a form of child abuse and, in some circumstances, can meet the definition of modern-day slavery and is commonly seen in youth gangs [2]. Safeguarding children and young people at risk of CCE, or gang involvement, is fundamental to protect the welfare, well-being, and safety of children and young people, families, and communities. There are few well-developed interventions to support this specific group of vulnerable children and young people. There are also significant challenges in engaging this group in interventions and research such as difficulties identifying children at risk of CCE; children not seeing themselves as being a victim of abuse; families being at crisis point and having little time to engage with services and research; families and young people distrusting new individuals entering the family system (i.e., practitioners, researchers) and fear of gangs/those who have exploited the young people seeking retribution. Developing and testing appropriate interventions and engagement methods for this group and their families, while finding effective and appropriate ways to engage this group in research, is critically important.

Multisystemic therapy (MST) is an intensive family-based intervention designed to support young people with antisocial behaviors. There is evidence that MST leads to a small but significant reduction in criminal offending behavior among children and adolescents [3]. Van der Stouwe et al. [3], in their meta-analysis of MST, reported that this effect was only present when participants were offenders and younger than 15 years; larger effect sizes were associated with the successful completion of treatment, and longer duration treatment. Further, effect sizes increased when the comparison group received a single and non-multimodel intervention as treatment-as-usual (TAU). Treatment effects were moderated by study characteristics, such as the study taking place in the USA, efficacy designs, and studies rated as higher quality. The effect on delinquency was moderated by changes in externalizing behavior, substance abuse, parenting skills and mental health, and placements away from home. Treatment for those with a history of sexual offending behavior was also associated with larger effect sizes.

It is of particular note that studies examining MST tend to report smaller effect sizes when located outside of the USA, for example, within Scandinavia [4–6] or Canada [7]. A recent Campbell Collaboration systematic review and meta-analysis [8] included 23 MST trials published between 1983 and 2020. They highlighted the inconsistency of study outcomes and findings, the mixed quality of the studies, and the high risk of bias in these studies. Overall, they found that, outside the USA, there is a lack of evidence that MST is effective.

In the United Kingdom, Butler et al. [9] completed a small randomized controlled trial of MST, reporting that the intervention led to improvements in recidivism over and above TAU within Youth Offending Teams in North London. This group went on to complete a large single-blind randomized controlled trial comparing MST to TAU within England [10], reporting that MST did not lead to a significant reduction in out-of-home placements, nor did it reduce the time to the next offense episode. The average number of offenses committed by participants at the 18-month follow-up was significantly greater for those who had received MST. Those who scored low on callous and unemotional traits had poorer outcomes at follow-up. The authors suggested that these outcomes may be associated with the relatively well-resourced nature of services for young offenders within the United Kingdom, including increased flexibility to respond to the treatment needs of young offenders, in comparison to the USA. A 5-year follow-up of the same families also found no difference between MST and TAU for offending behavior [11, 12]. However, a qualitative study interviewing these same young people 4 years after the completion of this trial found that the male participants who took part in MST had more mature profiles as they transitioned into adulthood than those who took part in TAU [13].

In order to deal with some of these challenges, a series of proposed augmentations to the standard MST protocol for children and teenagers who are at risk of criminal exploitation were proposed, which may increase the effectiveness, especially for those at risk of criminal exploitation. The proposed changes included:

- Extending the length of treatment to 4–6 months, which has been previously associated with larger effect sizes [3],
- The inclusion of psychoeducation about criminal exploitation,
- Expansion of the content of therapy to include a greater focus upon teaching coping skills, safety planning, trauma-focused work, substance misuse work, and addressing both distorted cognitions and social skills,
- Ensuring that the expectations and the content of any intervention are developmentally appropriate for children who are aged 10 to 12,
- The inclusion of setting clear expectations about curfews and peers,
- Targeting factors that lead to greater inclusion within the home environment, and reduce conflict between young people and adults,
- Increased supervision and monitoring of social media,
- Increased plans to retrieve a younger person if they go missing in collaboration with agencies (e.g., police),
- Increased safety planning around risky behaviors at home and in the community (e.g., weapons, aggression),
- Increased focus on de-escalation techniques to reduce family conflict,
- Increased focus on increasing pro-social peers and activities,
- Inclusion of training and resources for working with young people who are involved with risky adults/gangs, inclusive of mapping contacts with negative peer groups,
- Increased focus on the promotion of relationships with positive adults within the community,
- An increased focus on communication with parents,
- Working with primary schools, especially around periods of transition to secondary schools,
- Increased work to reduce exclusions and weapon carrying, and
- Increasing the focus on working collaboratively with agencies.

Taking the aforementioned literature and proposed augmentations to MST into account, the aim of the current project is to examine the feasibility of MST for children at risk of criminal exploitation, an augmented version of MST, adapted specifically for children and young people aged 10 to 15 years of age who are at risk of criminal exploitation. To achieve this, we will complete a modeling study within existing services to test the proposed changes to the standard MST protocol using process evaluation, and qualitative and quantitative research methods. The primary purpose of this methodology is to collect data to allow for the estimation of parameters necessary to inform the decision to proceed with a pilot clinical trial.

### Research questions

Our research questions are as follows:

Primary research question: (a) Is it feasible to complete a pilot trial of MST for children at risk of criminal exploitation within existing services within England?

Secondary research questions: (b) Do families, children and young people, clinicians, and other stakeholders consider MST for children at risk of criminal exploitation an acceptable intervention, and (b) what are the likely factors that will facilitate or hinder the successful implementation of MST for children at risk of criminal exploitation and how can they be successfully managed?

## Methods

### Study objectives

The objectives of this study are to:

- Collaboratively refine the logic model which represents the causal processes through which MST for children at risk of criminal exploitation leads to a reduction in criminal offending behavior. This logic model will be used to directly inform our process evaluation which will consider, but is not limited to (i) the impact of additional staff training, (ii) how partnerships with stakeholders are strengthened, (iii) the processes that promote or hinder greater engagement with voluntary and community agencies and other positive activities, and (iv) the processes that increase or hinder engagement in education and school transition.
- To undertake interviews with clinicians, stakeholders, families, and young people to refine MST tools for working with families where there is a risk of criminal exploitation, including adaptations to any existing fidelity checklists, and to consider the most appropriate method of measuring outcomes from MST for children at risk of criminal exploitation.
- To complete a single-group modeling study of MST, for children at risk of criminal exploitation, with 50 families receiving treatment within existing MST services, in order to estimate: (i) the acceptability and feasibility of MST for children at risk of criminal exploitation for stakeholders, including families, (ii) patient and clinician satisfaction with the intervention, (iii) the appropriateness of our measures in terms of their use within a future pilot trial, (iv) the appropriateness of an adapted fidelity checklist (if possible), (v) the accrual rate and willingness of teams to recruit participants, (vi) therapy completion rate and attrition, and (vii) the within-group effect size.

In addition, we will sample families, stakeholders, and clinicians to complete in-depth interviews as part of our process evaluation to further consider whether the augmentations to the current MST protocol for children at risk of criminal exploitation were successfully implemented, with reference to our logic model, and any associated factors that facilitated or hindered the successful implementation.

### Design

This is a pilot feasibility study and a process evaluation of MST adapted for children at risk of criminal exploitation. The project is composed of two phases with subphases and takes place over 24 months:

#### Phase 1

There were two complimentary workstreams within this Phase. Phase 1.1: We hosted three initial collaborative meetings between researchers and MST experts to finalize the logic model to be used in order to formalize the indicators that we will measure within our process evaluation. As discussed within our objectives, these will include, but are not limited to, (i) the impact of additional staff training, (ii) how partnerships with stakeholders are strengthened, (iii) the processes that promote or hinder greater engagement with voluntary and community agencies and other positive activities, and (iv) the processes that increase or hinder engagement in education and school transition. The purpose of these meetings, in addition to finalizing the logic model, was to consider the most valid method of measurement associated with each of our key indicators. Phase 1.2: We completed group and one-to-one interviews with clinicians, stakeholders, families, and young people with participants from across our four sites in England: (a) Birmingham (b) Nottingham (c) Yorkshire, and (d) Sandwell. The aims of this stage were to refine MST tools for working with families at risk of criminal exploitation, including adaptations to existing fidelity checklists, and to consider the most appropriate method of measuring outcomes from MST for children at risk of criminal exploitation. Within each group, the proposed changes to standard MST were discussed, along with selected outcome measures, and the MST Therapist Adherence Measure–Revised (TAM-R). Participants were asked to consider each proposed change, outcome measure, and the fidelity checklist in turn, and were encouraged to consider the likely benefits, disadvantages, and any associated implementation challenges. Each interview was recorded and transcribed. Transcriptions were analyzed using Framework Analysis [14]. The findings were considered by the study and delivery team collaboratively, responding to recommendations by making necessary changes as appropriate.

#### Phase 2

Within Phase 2, we are currently accruing participants for a single-group modeling study of MST for children at risk of criminal exploitation with 50 families within existing MST services to estimate the parameters necessary to inform the decision as to whether a pilot trial should be completed. We will examine (i) the acceptability and feasibility of MST for children at risk of criminal exploitation for stakeholders, including families, (ii) patient and clinician satisfaction with the intervention, (iii) the appropriateness of our measures in terms of their use within a future pilot trial, (iv) the appropriateness of an adapted fidelity checklist, (v) the accrual rate and willingness of teams to recruit participants, (vi) therapy completion rate and attrition, and (vii) the within-group effect size. We will also complete in-depth interviews with 12 families (6 who have successfully completed treatment, and 6 who have discontinued treatment but have consented to be interviewed) and 12 clinicians and stakeholders as part of the process evaluation, and to further investigate acceptability.

#### Phase 3

We will request anonymized data for all MST participants from the MST-I database. This database is located in the USA and contains data about all families who complete MST. These data will include demographic information (e.g., age, gender, ethnicity), treatment information (e.g., length of treatment, whether treatment was completed), and key outcome data (e.g., parent feedback about MST, whether the young person is in school, remained living in the family home, or has offended since taking part in MST, SDQ, if taken). Parents/carers consent to these data being added to the MST-I database and reported/analyzed anonymously as part of their MST participation agreement paperwork. Participating organizations have already collected and stored these data as part of their substantive roles. We will be paying the organization that runs the database (MST-I) an administrative fee to cover all of their costs related to producing and sending us an anonymized dataset. We will analyse and report these data.

### Randomization and blinding

As this is a feasibility study, using a single group of participants, randomization will not be tested. Testing randomization and the associated procedures would be completed within a future pilot trial which would aim to estimate the parameters necessary to inform the decision to undertake a Phase III clinical trial. However, as part of in-depth interviews with families and clinicians, willingness to be randomized to MST for children at risk of criminal exploitation + Treatment as Usual (TAU) vs. TAU will be investigated.

Considering that this is a feasibility study, masked assessors will not be used. However, again, as part of our in-depth interviews, how participants understand, and whether they accept masking, and the use of associated procedures to maintain masking, will be investigated with participants and clinicians.

### Study setting

**Fig. 1:**
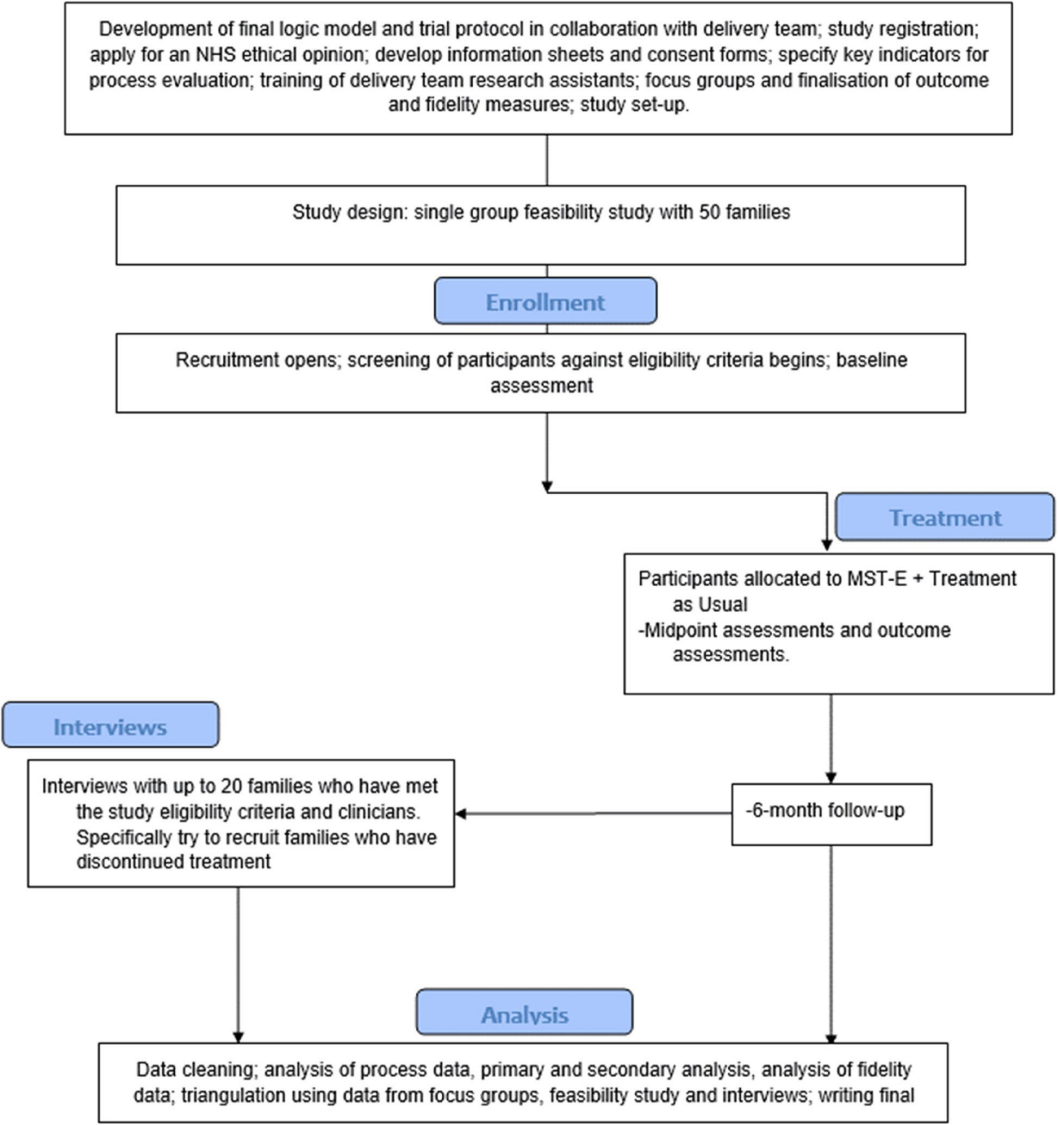
RESET Study flow diagram

This study is taking place within community-based settings in England. Sites are based in Birmingham, Sandwell, Yorkshire, and Nottingham, within existing MST services. Study sites have adopted a multi-agency approach to recruitment and invite referrals from social services, youth offending teams, child and adolescent mental health services, and education. The steps in the pathway for the pilot are as follows: families are informed about the screening process and if they do not opt-out, they are screened for eligibility to take part in MST for children at risk of criminal exploitation. Participants who meet these criteria are assigned to receive MST for children at risk of criminal exploitation and complete an initial baseline assessment within 4 weeks before starting MST treatment. MST for children at risk of criminal exploitation will last for up to 6 months, and an additional assessment period will occur at the mid-point of treatment (at 3 months, although sometimes shorter depending on the length of treatment), the end of treatment (approximately 6 months), and at 6-month follow-up. Young people, families, therapists, and key stakeholders will also be invited to take part in an interview as part of the process evaluation and our investigation of the acceptability and experience of treatment. Further information can be found in the participant flow diagram (Fig. 1).

### Fidelity and adherence

In collaboration with the delivery team and MST-UK, we will consider making appropriate augmentations to the existing MST instrument, the TAM-R [15–17] which is used routinely within MST as a measure of therapist fidelity. We will also record and report the number of sessions received, as well as the attrition rate.

### Inclusion criteria and exclusion criteria

We are currently in the process of recruiting 50 children and young people aged 10 to 15 years who have a history of engaging in antisocial behavior. Our eligibility criteria have been adapted from a successfully completed phase III trial of MST [10].

### Inclusion criteria

The inclusion criteria for pilot participants are as follows:

1. Children aged between 10 and 15 years
2. Parental consent to take part
3. The child is at risk of exploitation, as evidenced by:
  (A) Either the child or family having disclosed information to indicate that the child is at risk of another individual or group taking advantage of an imbalance of power to coerce, control, manipulate, or deceive them into any criminal activity in exchange for something the victim needs or wants, for the financial or other advantage of the perpetrator or facilitator or through violence or the threat of violence;
  (B) Or there is evidence of at least two of the following present which suggests that a child is at risk of exploitation:
    i. A criminal conviction, or a final warning, cautions, or reprimands within the last year,
    ii. Exhibiting weekly aggressive behavior which is of a significant risk to others (e.g., sexually abusive behavior, physical fighting) outside the home,
    iii. At least one period of having gone missing, even for a few hours, within the last 6 months,
    iv. History of substance misuse (alcohol or drugs),
    v. History of permanent school exclusion,
    vi. Association with peers or adults who are seen by others to have had a negative influence upon the child.

### Exclusion criteria

Our exclusion criteria for pilot participants are as follows:

1. The family refuses to take part in the study.
2. The young person lives independently, or a primary caregiver cannot be identified.
3. The child is presenting with symptoms consistent with a psychotic illness.
4. The child is at high risk of suicide as assessed by the clinical team assessing the referral.
5. There is documented evidence of the child having a Full-Scale IQ < 65.
6. There is evidence to indicate that a family member who is living with the child has been sexually abusing them and there continues to be an active and enduring risk,
7. The child has previously received a diagnosis of an autism spectrum disorder and problematic behaviors as defined within the inclusion criteria have been judged to be associated with having a developmental disability (e.g., self-harm associated with hypersensitivity) by the clinical team assessing the referral.

### Sampling

For phase 1 of this study, we worked collaboratively with the delivery team and their partner organizations to identify and invite potential participants to take part in our interviews. Phase 2: In order to maximize the probability of successful recruitment, we have adopted a multi-agency approach to recruitment and invite referrals from social services, youth offending teams, child and adolescent mental health services, and education. Fifty children and teenagers from one of three geographical regions in England (i.e., Birmingham and Sandwell, Nottingham, Yorkshire) who have met the eligibility criteria for the study, along with their families, will be invited to take part. As this is a feasibility study, and the purpose is to provide estimates of key parameters for a future pilot trial rather than to power the current study to detect statistically significant differences, a formal a priori power calculation has not been conducted [18]. However, recruiting 50 participants will provide a certain level of precision around a 95% confidence interval regarding the estimate of within-group effect. For example, the 95% confidence interval associated with a sample size of 50 is ± 13.8. We therefore believe that this sample size will provide reasonable precision around our estimates of parameters which will be used to inform the design and decision to proceed to a pilot trial including an estimate of the standard deviation of the outcome measures. We will also recruit 48 participants to take part in our interviews about the interventions which will contribute to the decision as to whether to proceed to a definitive trial. However, should a decision be made to proceed to a trial within a future study, it would be appropriate to recruit a larger sample than what we have proposed for inclusion within this feasibility study in order to model the parameters (e.g., randomization) necessary to inform the decision to proceed to a phase III trial.

### Data collection

Our proposed primary and secondary outcome measures are outlined below. These were discussed and considered collaboratively with MST professionals, and further reviewed by participants who took part in our phase 1.2 interviews. Eligible participants complete outcome measures 4 weeks before the commencement of treatment, and then at up to 13 (mid-point), up to 26 (end of treatment), and 52 weeks (12-month–6-month follow-up) from treatment commencing.

### Screening measures

Participants and their families will initially be invited to take part in a screening interview to ensure that they meet the eligibility criteria for the study, including the collection of evidence to confirm that a child is at risk of exploitation. Children and teenagers will be invited to complete the Wechsler Abbreviated Scale of Intelligence-II [19]. Referrers (e.g., youth offending teams, child and adolescent mental health teams) will be asked to confirm the presence of antisocial behavior.

### Candidate primary outcome measure

The candidate primary outcome measure is the Self Report Delinquency Measure (SRDM) [20] which was developed and used as part of a longitudinal study of criminal offending behaviors amongst 4300 children in Edinburgh. This is a short measure comprising 15 items pertaining to antisocial behaviors (e.g., burglary, violence) which require children to respond with yes or no with reference to a time period, and then report the estimated frequency of behavior, and whether they have ever been caught. There is evidence that asking respondents to indicate whether they have engaged in these behaviors is accurate [21, 22], and this measure has been previously used as an outcome measure within a randomized controlled trial of MST [10].

### Secondary outcome measures

Potential secondary outcome measures include (1) crime data: we will work with sites to gain information about arrests, cautions, reprimands, warnings, and convictions for participants with consent from parents. We aim to initially collect crime data over the prior 6-month period to the commencement of treatment, during treatment, and during the 6-month follow-up period. (2) Empathy: we will use the parental/carer version of the Griffith Empathy Measure (GEM) [23] which is a short 23-item measure of cognitive and affective empathy for children and adolescents which can be used with children aged from 4 years. The GEM has robust psychometric properties [23]. (3) Callous and unemotional traits: this will be measured using the 24-item Inventory of Callous and Unemotional Traits–Parent Report and Youth Self-Report Versions [24] which are robust and well-validated instruments [25]. (4) Well-being: the parent and self-report version of the Strengths and Difficulties Questionnaire (SDQ) will be used to assess child and teenager well-being. The SDQ is a robust and well-validated measure of behavioral and emotional problems [26]. (5) Peer Deviance: The Behavior of Friends Questionnaire (BFQ) [27] is a short 10-item checklist that assesses peer delinquency by asking respondents to indicate whether their friends engage in a variety of antisocial behavior (e.g., lying, stealing) using a 5-point Likert scale. The measure has been shown to have excellent internal consistency [27]. (6) Parenting: the Alabama Parenting Questionnaire has robust psychometric properties and measures parental involvement, positive parenting, monitoring/supervision, inconsistent discipline, and punishment. Both the parent and child-completed versions will be used [28, 29]. (7) Satisfaction: at the end of treatment, we will assess acceptability by asking all children, adolescents, and parents to complete a short questionnaire containing 10 items that are answered using a Likert scale. Items specifically ask about satisfaction with taking part in the intervention. (8) Social deprivation: we will draw on data from the UK Indices of Multiple Deprivation to identify families living in deprived neighborhoods according to participants’ postcodes (i.e., zip codes). (9) Family functioning: Family Adaptability and Cohesion Scales–IV (FACES-IV) is a 62-item self-report measure that assesses cohesion, adaptability, communication, and satisfaction within a family system [30]. (10) Gang affiliation: the Gang Affiliation Risk Measure (GARM) [31, 32] is a short 15-item measure of gang affiliation that was developed with teenagers and young people from England.

#### Phase 2.2: process evaluation

Initially, we refined a logic model together with MST experts which will be used to directly inform our process evaluation. This will consider but is not limited to (i) the impact of additional staff training, (ii) how partnerships with stakeholders are strengthened, (iii) the processes that promote or hinder greater engagement with voluntary and community agencies and other positive activities, and (iv) the processes that increase or hinder engagement in education and school transition. We will develop a series of process indicators drawn from the logic model that we will measure throughout the study. We have also completed interviews with clinicians, stakeholders, families, and young people to refine MST tools for working with families where there is a risk of criminal exploitation, and to consider the most appropriate method of measuring outcomes from MST for children at risk of criminal exploitation. In addition, we will recruit families (*n* = 12) and clinicians and stakeholders (*n* = 12) to complete in-depth interviews as part of our process evaluation to further consider whether the augmentations to MST for children at risk of criminal exploitation were successfully implemented, with reference to our logic model, and any associated factors that facilitated or hindered the successful implementation. We will also attempt to interview families who have dropped out of treatment in order to understand the reasons (*n* = 6) as part of the 12 families that we intend to recruit.

### Analysis

As this is a feasibility study, the quantitative analysis will be descriptive in nature. Continuous data will be reported as means and standard deviations, or medians and interquartile ranges, as appropriate. Categorical data will be reported as frequencies and proportions. All data will be reported both overall and by site including recruitment and retention rates, outcome measure completion rates, and missing data rates. Outcomes will be estimated with their associated 95% confidence intervals. The study will be reported in accordance with the CONSORT extension for randomized pilot and feasibility studies [35].

Interviews will be transcribed and analyzed using Framework Analysis [14] which is well suited to this context because we have specific questions, an awareness of potential issues, and a pre-designed sample. This method allows researchers to investigate key issues of interest, rather than analyze data for emergent themes including the acceptability of the intervention and research procedures. We will triangulate data by comparing data collected during the focus groups, the feasibility study, and the interviews with families and clinicians, highlighting areas of agreement and disagreement using a triangulation protocol, integrating our qualitative and quantitative findings, similar to previously used methods [33, 34]. Data from all sources will be analyzed independently, and then compared using three different types of triangulation: (a) methodological—using more than one method to collect data, (b) data—using multiple data sources, and (c) investigator—using multiple researchers to analyze the data.

The outcomes that will be analyzed in order to judge the feasibility of completing a larger study are: (a) the acceptability and feasibility of MST-E for stakeholders, including families, including a description of factors that facilitate or hinder the implementation of the intervention, (b) the appropriateness of our measures in terms of their use within a future pilot trial including completion and missing data rates, (c) the appropriateness of an adapted fidelity checklist, if used, (d) the accrual and retention rate and willingness of teams to recruit participants, (e) therapy completion rate and attrition, and (f) the within-group effect size on our proposed primary and secondary outcome measures.

### Progression criteria

Green: (a) intervention acceptability: all stakeholders, including families judge the intervention and the associated changes from standard MST to be acceptable, (b) candidate outcome measures: 95% of recruited participants complete the outcome measures on all occasions and rates of missing data are below 5%, (c) fidelity checklist: an adapted fidelity checklist is judged to be acceptable by stakeholders with a completion rate of 95%, and (d) recruitment and retention: 100% of participants are recruited, the accrual rate is 2:1, and the retention rate is at least 80%.

Amber: (a) intervention acceptability: some stakeholders, including families raise concerns about the acceptability of the intervention, which may include confusion about how the intervention is sufficiently different from standard MST, (b) candidate outcome measures: fewer than 95% but more than 80% of recruited participants complete the outcome measures on all occasions and rates of missing data are more than 5% but less than 10%, (c) fidelity checklist: an adapted fidelity checklist is judged to be acceptable by stakeholders with a completion rate of less than 95% but more than 80%, and (d) recruitment and retention: fewer than 100% but more than 80% of participants are recruited, the accrual rate is greater than 2:1 but less than 2.5, and the retention rate is less than 80% but higher than 70%.

Red: (a) intervention acceptability: a majority of stakeholders, including families raise concerns about the acceptability of the intervention, including confusion about how the intervention is sufficiently different from standard MST, (b) candidate outcome measures: fewer than 80% of recruited participants complete the outcome measures on all occasions and rates of missing data are more 10%, (c) fidelity checklist: an adapted fidelity checklist is judged to be acceptable by stakeholders with a completion rate of less than 80%, and (d) recruitment and retention: fewer than 80% of participants are recruited, the accrual rate is greater than 2:5, and the retention rate is less than 70%.

Where any of our criteria are judged to be amber or red, the study team will examine the likely causes and consider whether a mitigation strategy could be successfully implemented.

Where any of our criteria are judged to be amber or red, the study team will examine the likely causes and consider whether a mitigation strategy could be implemented.

### Ethics

This study received NHS Health Research Authority approval, having been reviewed and given favorable opinion by Yorkshire & The Humber-South Yorkshire Research Ethics Committee (3rd December 2020). All participants have/will be provided with study information and will sign an informed consent form. Parental consent is required for the inclusion of participants under the age of 16, while agreement from each child will also be sought. Information about the study is provided to parents and children, and this information was adapted for use by younger children to help ensure that information about the study is accessible. Where parents have difficulties with reading and writing, we will provide study information in an alternative format (e.g., audio file). We will also provide translations of study documents and interpreter services to include families who do not speak English. Parents will have the right to withdraw their child from the pilot. We will abide by the General Data Protection Regulations (2018). All data will be stored securely on university servers. All data will be confidential, and it will not be possible to identify a child or any member of their family within any publication arising from this work. All incidents and near misses will be reported to the University of Warwick Health and Safety Department via the Accident, Incident, and Near Miss Reporting Form.

## Results

Figure 1 provides the participant flow diagram. Accrual to the pilot stage of this project opened on 6th August 2021 and is due to close on 31st May 2022. We aim to publish the results of this feasibility study and process evaluation in 2023.

## Discussion

In this protocol, we have summarized a feasibility study and process evaluation for MST adapted specifically for children at risk of criminal exploitation and their families. This is an important group of young people to safeguard and support. Our primary aim is to understand whether it is feasible to complete a pilot trial of MST for children at risk of criminal exploitation, within existing services in England. Our secondary aims are to understand the acceptability of MST for children at risk of criminal exploitation, as well as to understand factors that will facilitate or hinder the successful implementation of MST for children at risk of criminal exploitation, and how they can be successfully managed. In terms of the current study status (May 2022), we have completed the adaptations to the logic model (phase 1.1) and the pre-pilot interviews (phase 1.2). We are in the final month of accruing new participants to the study pilot and we will continue to follow-up with these families until April 2023 (phase 2.1). Finally, we are about to commence accrual to the process evaluation/post-treatment interviews (phase 2.2).

In conclusion, the results of this study will inform whether it is advisable to progress onto a definitive trial for this adaptation of MST for children at risk of criminal exploitation. These young people and families are under-researched and hard to engage. Therefore, the lessons learned during this study about recruiting and retaining these families in research will inform future work with this population. Findings about the adaptations made to this research, and to the intervention during the COVID-19 pandemic will also provide useful lessons to inform future research.

## Data Availability

Once the study has been completed and the findings have been reported pilot data will be submitted to the Department for Education (UK). These data will then be anonymized and submitted to the Office for National Statistics Secure Research Service (UK). Once available, restrictions will apply to the access of these data to protect study participants. This process is in line with our funding requirements from the Youth Endowment Fund. Participants have consented to their data being used in this way.

## Abbreviations

MST: Multisystemic therapy
CCE: Child criminal exploitation
TAU: Treatment as usual
